# The Association between Purine-Rich Food Intake and Hyperuricemia: A Cross-Sectional Study in Chinese Adult Residents

**DOI:** 10.3390/nu12123835

**Published:** 2020-12-15

**Authors:** Sumiya Aihemaitijiang, Yaqin Zhang, Li Zhang, Jiao Yang, Chen Ye, Mairepaiti Halimulati, Wei Zhang, Zhaofeng Zhang

**Affiliations:** 1 Department of Nutrition & Food Hygiene, School of Public Health, Peking University Health Science Center, Haidian District, Beijing 100191, China; 1410606101@pku.edu.cn (S.A.); zhangyaqin@bjmu.edu.cn (Y.Z.); yjiao@bjmu.edu.cn (J.Y.); 1510306235@pku.edu.cn (C.Y.); 2011210145@bjmu.edu.cn (M.H.); zhangwei1993@bjmu.edu.cn (W.Z.); 2 Department of Population Health Sciences, School of Population Health & Environmental Sciences, King’s College London, London SE1 1UL, UK; li.1.zhang@kcl.ac.uk

**Keywords:** purine-rich food, hyperuricemia, serum uric acid, animal-derived food

## Abstract

Objective: To explore the correlation between purine-rich food intake and hyperuricemia in Chinese adult residents. Method: A cross-sectional study was conducted on the purine-rich food intake of Chinese adult residents based on the China Health and Nutrition Survey (CHNS) in 2009. The subjects were divided into hyperuricemia group and nonhyperuricemia group according to serum uric acid level, and the differences of the sociodemographic information (age, gender, and region), health status (weight status, blood pressure, blood sugar status), living habits (alcohol consumption, smoking status) and food intake (purine-rich food, other food) were compared between the two groups. Logistic regressions investigated the associations between the daily intake of purine-rich food (animal-derived food and legumes) and hyperuricemia. Results: Eventually, 6813 subjects were included in our study, 1111 of them had hyperuricemia. The intake of seafood, legumes, red meat, and poultry all increased the risk of hyperuricemia (*p* < 0.05), while the intake of purine-rich fungi and purine-rich vegetables did not affect the occurrence of hyperuricemia. Animal-derived food was the main source of purine-rich food consumed by Chinese adult residents (140.67 g/day), which had a great impact on hyperuricemia. Finally, after adjusting for gender, age, region, body mass index (BMI), alcohol consumption, hypertension, and refined grains intake, the risk of hyperuricemia increased by 2.40% and 1.10% for each increase of 10 g in animal-derived food intake (OR = 1.024, 95% CI: 1.018–1.030) and legumes intake (OR = 1.011, 95% CI: 1.003–1.019), respectively. Conclusion: The intake of animal-derived food and legumes were positively correlated with the occurrence of hyperuricemia. Controlling the intake of animal-derived food and legumes would be more beneficial to controlling the risk of hyperuricemia.

## 1. Introduction

Hyperuricemia occurs due to the urate overproduction or impaired urate excretion through the kidney and gastrointestinal tract [1]. Hyperuricemia is considered to be a precursor of gout as the deposition of urate crystals in the joints results in an acute inflammatory response. Deposition in the soft tissue can lead to tophi [2,3]. Gout is also a serious health issue and is an independent risk factor for heart failure and metabolic syndrome [4,5]. Acute gout is a painful crippling state, and left untreated, chronic gout can lead to chronic joint pain, joint erosion, injury, and form gout nodule, which can rupture, infect or cause other complications, such as hypertension [6,7], chronic kidney disease [8,9], obesity, diabetes [10], myocardial congestion, stroke [11,12]. Besides, hyperuricemia is associated with chronic kidney disease (CKD), cardiovascular disease, hypertension, diabetes, metabolic syndrome, and obesity [13,14,15,16,17].

It is reported that the prevalence of gout and hyperuricemia is increasing globally, which has brought a huge burden to the human body and society, and both diseases have become public health problems that need to be solved quickly [2,18,19,20]. Especially in developed countries, the prevalence of gout is 3–6% in males and 1–2% in females [21,22]. According to the National Health and Nutrition Examination Survey of the United States, the total prevalence of gout and hyperuricemia was 3.9% and 21.4% [19]. According to the UK clinical practice research database, the prevalence of gout was 2.5% [5]. The reported prevalence rate of hyperuricemia ranges from 8.9% to 24.4% [19,23,24] in different populations. As the largest developing country, according to the systematic review and meta-analysis, the pooled prevalence of hyperuricemia was 13.3% (95% CI: 11.9%, 14.6%), with the prevalence ranging from 5.5% to 23.6%. The pooled prevalence of gout was 1.1% (95% CI: 0.7%, 1.5%), with a range of 0.4 to1.5% [25]. 

Although the risk factors for hyperuricemia have not been fully determined, recent studies have shown that apart from genetic variations [26], several lifestyle or dietary factors have been associated with hyperuricemia. As uric acid is an ultimate product of purine metabolism, hyperuricemia is related to the intake of purine-rich food. However, studies linking food intake with uric acid or hyperuricemia are limited and the latest evidence suggested that not all purine-rich food affects uric acid levels in the same manner. With the development of the economy and the change of lifestyle, the dietary pattern has also changed. The intake of seafood, red meat, poultry, and legumes has increased gradually. It has been found that purine content is high in most seafood, red meat, poultry, legumes, and a few vegetables and fungi [27,28,29,30]. According to the previous research results, it seems that the purine-rich food of meat origin is related to the risk of gout, whereas the purine-rich food of plant origin is not as effective as that of meat origin [31,32,33,34]. Although there are relevant reports in other countries, people’s sensitivity to food will be different due to different races and genes. It is impossible to directly use the research results of other countries to speculate on the situation in China.

At present, the research on purine-rich food intake of Chinese residents in recent years is very limited. Although there are some relevant studies in China, the population range is relatively small and limited, and the results of these studies have limited suggestions on Chinese dietary intake. Besides, the guidelines for the diagnosis and treatment of hyperuricemia in China also contain recommendations to limit the intake of purine-rich food. However, purine-rich food is a big concept and general term which contains too many foods. Whether all purine-rich food is harmful remains to be determined. 

Therefore, we conducted a cross-sectional study based on the China Health and Nutrition Survey (CHNS) to explore the correlation between hyperuricemia and purine-rich food intake in the Chinese population, and the findings will ultimately be a guide to prevent hyperuricemia and gout from dietary factors.

## 2. Materials and Methods

### 2.1. Data Collection and Samples

This cross-sectional study is based on CHNS. CHNS is an ongoing open cohort, an international collaborative project between the Carolina Population Center at the University of North Carolina at Chapel Hill and the National Institute for Nutrition and Health (NINH, former National Institute of Nutrition and Food Safety) at the Chinese Center for Disease Control and Prevention (CCDC). This study covers 15 provinces and cities, which is representative of Chinese people and can better reflect the nutritional and health status of the Chinese people. The first CHNS survey was completed in 1989, followed by surveys every two or three years, and ten surveys have been completed so far. In our study, we used the results of the 2009 survey, because blood sampling information was collected only in 2009.

Our study content includes the health status, nutrition status, life behavior, and general demographic information of Chinese residents extracted from CHNS. The health status information is collected by physical examination and inquiry. The personal dietary data is collected by a 3-day 24-h meal review method. Life behavior and general demographic information are collected by the questionnaire survey.

**Figure 1:**
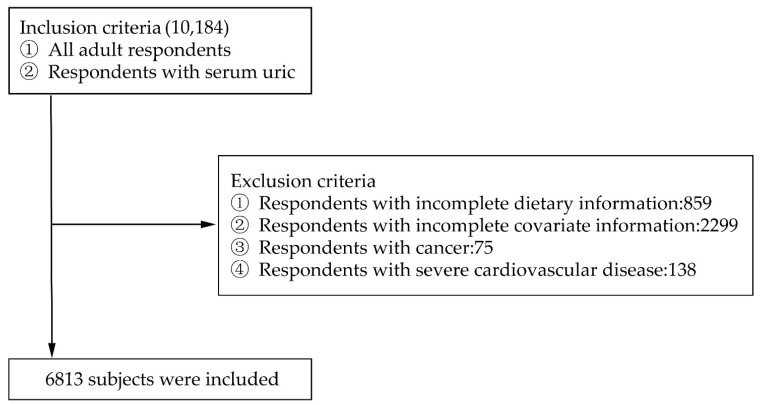
Flow chart for inclusion and exclusion of research subjects.

All adult respondents with serum uric acid index in the 2009 survey of CHNS were included in the study, and those with incomplete dietary information, pregnancy, cancer, and severe cardiovascular disease were excluded. 6813 subjects were finally included in the study (Figure 1).

All subjects gave their informed consent for inclusion before they participated in the study. The study was approved by the Institutional Review Board of the University of North Carolina at Chapel Hill and the National Institute for Nutrition and Health, Chinese Center for Disease Control and Prevention (2015017).

### 2.2. Definition of Hyperuricemia

In this study, according to the Definition of hyperuricemia and gouty conditions and Consensus of multidisciplinary experts on diagnosis and treatment of hyperuricemia related diseases in China [35,36], Subjects with UA ≥ 420 μ mol/L (7 mg/dL) for men or 360 μ mol/L (6 mg/dL) for women were determined as hyperuricemia and assigned to the hyperuricemia group, and the rest were automatically assigned to the nonhyperuricemia group. 

### 2.3. Definition and Evaluation of Purine-Rich Food

According to the purine content and classification of various foods in the article published by Pan Hongzhi and Rong Shengzhong [27,28,29,30], in our study, foods with purine content ≥1000 mg/kg were defined as purine-rich foods, including red meat, poultry, seafood (fish and shellfish), legumes, purine-rich vegetables (spinach, cauliflower, laver, *Auricularia auricula*, asparagus, celery, coriander) and purine-rich fungi (shiitake mushroom, *Agrocybe aegerita*, *Pholiota nameko*, *Pleurotus ferulae* Lanzi, hazelnut mushroom, *Hericium erinaceus*) [27,28,29,30]. The average daily intake of various purine-rich foods and animal-derived purine-rich food were determined as independent variables.

### 2.4. Assessment of Covariates

According to the conclusion of previous relevant studies, confounding factors: BMI, smoking status (yes/no), alcohol consumption (yes/no), hypertension (yes/no), diabetes (yes/no), other food intakes, and general demographic information: age, gender, region, and educational level were included in the study. 

### 2.5. Statistical Analysis

The subjects were divided into hyperuricemia group and nonhyperuricemia group. Independent Chi-square test was used to examine the basic characteristics of the two groups, such as gender, age, region, education, BMI, alcohol consumption, hypertension, diabetes, and smoking status. Non-parametric tests were performed on the continuous variables of non-normal distribution between the two groups, such as purine-rich food intake and other food intakes. We judged the covariates that were eventually included in the study based on whether the basic characteristics of the hyperuricemia group and the nonhyperuricemia group were different.

To examine the associations between dietary purine-rich food intake and hyperuricemia, we constructed multilevel logistic regression models. First, univariate regression was performed for each purine-rich food intake (seafood, legumes, red meat, poultry, purine-rich fungi, and purine-rich vegetables) and hyperuricemia. On this basis, it was mainly analyzed in three models: original model univariate logistic regression models for purine-rich food intake (animal-derived food and legumes) and hyperuricemia without any adjustments, model 2 adjusted for dark vegetable (Dark vegetables refer to dark green, red, orange, and purple vegetables. Their leaves or fruits are often darker in color, and they are rich in calcium, iron, and vitamin B2.) intake and refined grains intake, model 3 additionally adjusted for gender, age, region, education level, BMI, alcohol consumption, smoking status, hypertension, and diabetes.

Interaction analyses were conducted to evaluate whether the association observed between animal-derived food intake and the risk of hyperuricemia could be modified by dark vegetable intake (≤100.0 g, >100.0 g), and refined grains intake (≤346.7 g, >346.7 g). 

Data preprocessing, database establishment, and statistical analysis were all completed by SAS University edition software (Copyright © 2012–2020, SAS Institute Inc., Cary, NC, USA), unless otherwise indicated. (*p* < 0.05 was used as the statistical index of significance test).

## 3. Results

### 3.1. Baseline Characteristics

6813 subjects were included in this study. The number of women (3595, 52.77%) was slightly higher than that of men (3218, 47.23%). The rural residents (4586, 67.31%) were more than the urban residents (2227, 32.69%). More than half of the subjects’ weight was in the normal range (3643, 53.47%), the emaciated (439, 6.44%) were far less than the overweight (2731, 40.09%). The total number of people with a smoking history was 2128 (31.23%), and most of the subjects never smoked (4685, 68.77%). There were some subjects (2095, 30.75%) suffered from hypertension; a few subjects (210, 3.08%) suffered from diabetes (Table 1).

More than 75% of the subjects consumed more than 30.00 g/d of red meat, more than half of the subjects consumed more than 35.00 g/d of legumes, and 75% of the subjects did not eat purine-rich fungi and purine-rich vegetables. Only 25% of the subjects consumed 5.00 g/d of wholegrains, while refined grains intake was the most that more than 25% of the subjects consumed more than 250.00 g/d.

In this study, there were 1111 (16.31%) subjects with hyperuricemia: males (676, 60.85%) were more than females (435, 39.15%). There were differences (*p* < 0.05) in gender, age, region, education level, BMI, smoking status, alcohol consumption, hypertension, and diabetes between the two groups (Table 1). The intake of seafood, red meat, poultry, legumes, and purine-rich fungi was different (*p* < 0.05), whereas the intake of purine-rich vegetables, was not statistically different; the intake of dark vegetables and refined grains was different (*p* < 0.05), other intakes were not statistically different (Table 2).

### 3.2. The Association of Dietary Purine-Rich Food Intake and Hyperuricemia

According to Table 2, we found that intake of seafood, purine-rich fungi, legumes, red meat, and poultry were different between the two groups (*p* < 0.05), While there is no statistical difference between the two groups in terms of the intake of purine-rich vegetables (*p* > 0.05). Multivariate logistic regression analysis was used to analyze the intake of these five kinds of food. We found that the intake of seafood, legumes, red meat, and poultry had an impact on the risk of hyperuricemia (*p* < 0.05), while the intake of purine-rich fungi had no effect on the occurrence of hyperuricemia (Table 3).

Seafood, red meat, and poultry were animal-derived foods. Therefore, the intake of animal-derived food and legumes were analyzed by model 1, 2, and 3, respectively. Logistic regression analysis showed that animal-derived food intake and legumes intake were associated with the risk of hyperuricemia in the three models (Tables S1–S3). After adjustment (model 3), the OR of animal-derived food intake was 1.024 (95% CI: 1.018–1.030), indicating that the risk of hyperuricemia increased by 2.40% for every 10 g of animal-derived food intake, and 1.011 (95% CI: 1.003–1.019) for legumes intake, indicating that the risk of hyperuricemia increased by 1.1% for every 10 g legumes intake. Model 3 has a lower OR than model 1, indicating that their influence has been overestimated before (Table 4).

Besides, the results of the multiple logistic regression analysis showed that gender, age, BMI, region, alcohol consumption, hypertension, and intake of refined grains all influenced the occurrence of hyperuricemia (*p* < 0.05). Males were more likely to suffer from hyperuricemia (OR = 1.772, 95% CI: 1.465–2.143). The risk of hyperuricemia in the people age <45 was 80.6% of those age ≥60 (OR = 0.806, 95% CI: 0.659–0.985), the risk of hyperuricemia in the people 45 ≤ age < 60 was 80.6% of those age ≥60 (OR = 0.806, 95% CI: 0.677–0.959). The risk of hyperuricemia of residents in rural areas was 79.9% , and in urban areas (OR = 0.799, 95% CI: 0.690–0.926). The risk of hyperuricemia for BMI <18.5 was 31.7% for those BMI ≥24 (OR = 0.317, 95% CI: 0.219–0.460), The risk of hyperuricemia for 18.5 ≤ BMI < 24 was 51.6% for those BMI ≥24 (OR = 0.516, 95% CI: 0.449–0.594). Alcohol consumption increased the risk of hyperuricemia (OR = 1.221, 95% CI: 1.035, 1.422). Having hypertension increased the risk of hyperuricemia (OR = 0.572, 95% CI: 0.493, 0.664). The OR of refined grains intake in covariate was 0.991 (95% CI: 0.986–0.995), which indicated that the intake of refined grains was the influencing factor of hyperuricemia. The risk of hyperuricemia would be reduced by 0.90% for every 10 g of refined grains intake increased (Table S3).

### 3.3. Interaction of the Effect of Animal-Derived Food Intake on Hyperuricemia

According to Table 2, we found that dark vegetable intake and refined grains intake were different in the hyperuricemia group and the nonhyperuricemia group. Based on this difference, we conducted interaction analyses and found that dark vegetable intake and refined grains intake had no interaction with animal-derived food intake in terms of the impact on hyperuricemia (There was neither a multiplicative interaction nor additive interaction, Tables S4 and S5).

## 4. Discussion

Our results showed that a higher intake of animal-derived food and legumes were associated with a higher risk of hyperuricemia in this cross-sectional analysis of 6813 adult participants. Those associations were independent of socioeconomic and lifestyle factors frequently associated with intake of purine-rich food and hyperuricemia, such as dietary intake of refined grains, gender, age, region, BMI, alcohol consumption, and hypertension. 

The relationship between diet and hyperuricemia has attracted a lot of attention. Some studies have shown that the lifestyle of the subjects has a significant impact on the occurrence of hyperuricemia, such as eating habits, as well as some demographic characteristics [37,38]. Several studies have shown that both meat and seafood intake are associated with hyperuricemia. In a study conducted in five coastal cities in China, hyperuricemia was associated with increased consumption of meat, fish, and shellfish [39]. A U.S. study of 14,809 subjects from the third National Health and Nutrition Examination Survey (NHANES-III) reported that serum uric acid levels increased with meat and seafood intake increased [30]. Meanwhile, studies have shown that eating purine-rich vegetables did not increase the risk of gout [32,40] and hyperuricemia. Contrarily, the level of serum uric acid decreased in vegetarians and spinach supplementation [41]. We did not find any studies that, in the long term, showed a significant positive association of purine-rich vegetables intake and urate. In our study, it was found that the animal-derived food intake was associated with the risk of hyperuricemia, whereas no association between purine-rich fungi, purine-rich vegetables and the risk of hyperuricemia were found, which was consistent with these reports and the results of previous studies. The guidelines for the diagnosis and treatment of hyperuricemia in the United States and Europe suggest that the intake of animal-derived food is restricted, but the intake of purine-rich vegetables and purine-rich fruits is not restricted, which is similar to our results. We believe that both the U.S. and European guidelines and our findings have reference significance for the diagnosis and treatment of hyperuricemia in our country. We can consider limiting the intake of animal-derived food, but not limiting the intake of plant-derived food as a treatment recommendation.

A recent study has shown that in individuals with metabolic syndrome, total non-soy legume, lentil, and pea consumption were inversely associated with SUA levels. Moreover, non-soy legumes and their different subtypes, except for chickpeas, were associated with a lower prevalence of hyperuricemia [42]. The study showed that despite being considered a purine-rich food, legumes contain other nutrients that could exert a beneficial effect on uric acid levels. This conclusion was inconsistent with our conclusion that legumes intake was associated with the risk of hyperuricemia. We considered this may be related to the inconsistency of the specific foods contained in legumes in each study. In addition, soya milk is a very common and unique drink in China. In our study, the consumption of soya milk may be directly converted into the consumption of legumes because it is not possible to calculate accurately at the data collection stage, this may lead to the calculated intake of legumes higher than the actual intake, while soya milk is preferred by older people, which may directly lead to higher legumes intake in older people than the actual intake. Besides, the amount of legumes intake and the way of cooking varies from region to region, which may cause differences in results.

Interestingly, in our study, the dark vegetable intake was different between the hyperuricemia group and the nonhyperuricemia group, but there was no effect on hyperuricemia in logistic regression analysis. This may be related to the intake of refined grains, which was far more than that of dark vegetables so that the impact of dark vegetables was reduced. The refined grains intake was the influencing factor of hyperuricemia, and the risk of hyperuricemia would be reduced by 0.90% for every 10 g of refined grains intake increased, and this effect still existed after adjustment. There was no clear study on the independent relationship between refined grains and hyperuricemia, but some studies have shown that the high blood pressure prevention plan like the DASH diet is related to a lower risk of a gout attack, while the Western diet is related to an increased risk of gout. In the study, the Western diet included a large intake of refined grains [43]. In our study, refined grains intake were the protective factors of hyperuricemia, which may have a lot to do with Chinese eating habits. Considering that there is no uniform dietary pattern for the subjects in this study, based on the common eating habits, refined grains is the main food of Chinese people. Because the intake of a meal is limited, the intake of a large amount of refined grains will reduce the intake of other foods, such as purine-rich foods. Therefore, refined grains indirectly become a protective factor. However, this is just a conjecture, and more research is needed to verify the relationship between the refined grains intake and hyperuricemia.

In our study, gender was the influencing factor of hyperuricemia. More males suffered from Hyperuricemia than females, which was consistent with the prevalence of hyperuricemia found in some previous studies [44]. This may be related to the fact that estrogen can promote the excretion of uric acid and inhibit the onset of arthritis in females [22]. The risk of hyperuricemia in male is 1.772 times higher than that in the female. The risk of hyperuricemia in the group age <45 and the group 45 ≤ age < 60 was respectively 80.6% and 80.6% of those age ≥60, which was consistent with the results that age and gender are the main risk factors for hyperuricemia and gout [45]. In our study, alcohol consumption was the influencing factor and increased the risk of hyperuricemia. This may be related to the fact that alcohol contains purine and contributes to the production of uric acid precursors and may also interfere with the excretion of uric acid [46]. Increasing studies have confirmed that hypertension and obesity were the risk factors of hyperuricemia. In 1997, Li et al. reported a population study from Beijing, which showed that serum uric acid concentration was closely related to triacylglycerol and glucose concentration and BMI [47]. A cross-sectional population study of 910 males and 603 females in Hong Kong showed that serum uric acid was positively correlated with BMI, waist-hip ratio, systolic and diastolic blood pressure, fasting blood glucose, triacylglycerol and apolipoprotein B, and negatively correlated with high-density lipoprotein cholesterol [48]. The authors suggested that serum uric acid may be a marker of cardiovascular disease risk, which was associated with hypertension, hyperlipidemia, and diabetes. The results of our study were consistent with those of these studies.

The strengths of this study include the sample size, adjustments for important confounders, such as the intake of other food, and population-based design. The data comes from CHNS, covering 15 provinces or municipalities. These areas are different in geographical location, economic development level, public resources, and health indicators. The survey adopts Multi-level cluster random sampling in each province or municipality and is more representative for the Chinese residents. In this study, according to the purine content of various foods in the article published by Pan Hongzhi et al. of the Harbin Medical University of China in 2012, calculating the purine-rich food intake of Chinese adult residents in the survey in 2009, is more scientific and objective, and more in line with Chinese eating habits. 

Several methodological limitations should be considered when interpreting the results of our study. A main limitation of the study was the outcome variable, hyperuricemia, which was defined only based on one blood test index of the study population, and no relevant medical diagnosis was obtained through a questionnaire or interview. According to the guidelines, the diagnosis of hyperuricemia requires two determinations of serum uric acid levels at different times. Because of objective factors, we did not know whether the subjects have taken medicine, so the results of serum uric acid may not represent the true situation. Therefore, we may underestimate the occurrence of hyperuricemia. Another main limitation needs to be mentioned that in our study, the purine content of each food is not complete in the classification of purine-rich food. Therefore, for the food without a specific value of purine content, it was classified according to the food with data in similar categories. Furthermore, this study is a cross-sectional study, and no causal inference can be made based on the results of this study. Additionally, although various influencing factors have been considered as comprehensively as possible, and as many covariates have been included as possible when the data allows, there are still many key factors that cannot be included in this study due to objective conditions(1. Endogenous purine metabolic disorders may also cause Hyperuricemia, but no relevant data have been collected; 2. Physiological renal dysfunction due to aging will lead to the reduction of secondary uric acid excretion, which may lead to hyperuricemia. However, the data collection of physiological renal dysfunction is incomplete, and so on).

## 5. Conclusions

In conclusion, we documented that a higher intake of purine-rich food was associated with a higher risk of hyperuricemia in China. Controlling the intake of animal-derived food and legumes would be more beneficial to controlling the risk of hyperuricemia, which may be a guide to prevent hyperuricemia and gout from dietary factors.

## Figures and Tables

**Table 1:** Characteristics of hyperuricemia group and nonhyperuricemia group (*n* = 6813).

Variable	Total (Number, %)	Hyperuricemia (Number, %)	Nonhyperuricemia (Number, %)	*χ* ^2^	*p*
Gender					
Male	3218 (47.23)	676 (60.85)	2542 (44.58)	98.700	<0.0001
Female	3595 (52.77)	435 (39.15)	3160 (55.42)		
Age (years)					
<45	2370 (34.79)	311 (27.99)	2059 (36.11)	39.581	<0.0001
45–59	2479 (36.39)	403 (36.28)	2076 (36.41)		
≥60	1964 (28.83)	397 (35.73)	1567 (27.48)		
Region					
Urban areas	2227 (32.69)	442 (39.78)	1785 (31.30)	30.383	<0.0001
Rural areas	4586 (67.31)	669 (60.22)	3917 (68.70)		
Education level					
None	1588 (23.31)	260 (23.40)	1328 (23.29)	11.762	0.0382
Grad from primary	1315 (19.30)	203 (18.27)	1112 (19.50)		
Lower middle school degree	2278 (33.43)	345 (31.05)	1933 (33.90)		
Upper middle school degree	818 (12.01)	140 (12.60)	678 (11.89)		
Technical or vocational degree	494 (7.25)	97 (8.73)	397 (6.96)		
University or college degree	320 (4.70)	66 (5.94)	254 (4.46)		
BMI (kg/m^2^)					
<18.5	439 (6.44)	34 (3.06)	405 (7.10)	158.564	<0.0001
≥18.5 & <24	3643 (53.47)	447 (40.23)	3196 (56.05)		
≥24	2731 (40.09)	630 (56.71)	2101 (36.85)		
Alcohol consumption					
No -drinker (1 year or above)	4539 (66.62)	631 (56.80)	3908 (68.54)	57.649	<0.0001
Current drinker	2274 (33.38)	480 (43.20)	1794 (31.46)		
Hypertension					
Patient	2095 (30.75)	509 (45.81)	1586 (27.81)	141.470	<0.0001
Nonpatient	4718 (69.25)	602 (54.19)	4116 (72.19)		
Diabetes					
Patient	210 (3.08)	56 (5.04)	154 (2.70)	17.039	<0.0001
Nonpatient	6603 (96.92)	1055 (94.96)	5548 (97.30)		
Smoking status					
Nonsmoker	4685 (68.77)	675 (60.76)	4010 (70.33)	39.649	<0.0001
Smoker	2128 (31.23)	436 (39.24)	1692 (29.67)		

BMI: body mass index.

**Table 2:** Food intake of hyperuricemia group and nonhyperuricemia group (*n* = 6813).

Dietary Factors	Intake (Averages, g/Day)			*p*
	All	Hyperuricemia	Nonhyperuricemia	
Purine-rich food				
Red meat	83.92	98.03	81.17	<0.0001
Legumes	62.37	70.79	60.72	0.0002
Seafood	38.31	47.80	36.46	<0.0001
Poultry	18.34	21.70	17.69	0.0017
Purine-rich vegetables	11.12	9.97	11.34	0.3695
Purine-rich fungi	2.32	3.14	2.16	0.0083
Other food				
Dark vegetables	118.88	122.77	118.13	0.0091
Other vegetables	212.18	212.87	212.04	0.5814
Fruits	52.54	53.84	52.29	0.2435
Refined grains	369.95	359.74	371.93	0.0272
Wholegrains	15.71	14.27	15.99	0.0670
Tubers	28.89	26.73	29.31	0.1233

**Table 3:** logistic regression analysis of various purine-rich food intake and hyperuricemia.

Variable	Intake (g/Day)		OR, 95% CI (10^−1^g/Day)	*p*
	Hyperuricemia	Nonhyperuricemia		
Seafood	47.80	36.46	1.022 (1.013, 1.032)	<0.0001
Purine-rich fungi	3.14	2.16	1.039 (0.990, 1.090)	0.1202
Legumes	70.79	60.72	1.014 (1.006, 1.022)	0.0003
Red meat	98.03	81.17	1.028 (1.019, 1.036)	<0.0001
Poultry	21.70	17.69	1.019 (1.004, 1.035)	0.0156

OR: Odds ratios; 95%CI: 95% confidence intervals.

**Table 4:** Odds ratio (95% CI) for hyperuricemia associated with animal derived food intake or legumes intake.

Variables	OR, 95% CI (10^−1^g/Day)	*p*
Animal-derived food		
Model1	1.025 (1.019, 1.030)	<0.0001
Model2	1.025 (1.019, 1.031)	<0.0001
Model3	1.024 (1.018, 1.030)	<0.0001
Legumes		
Model1	1.014 (1.007, 1.022)	0.0003
Model2	1.015 (1.008, 1.023)	<0.0001
Model3	1.011 (1.003, 1.019)	0.0080

OR: Odds ratios; 95%CI: 95% confidence intervals.

## Supplementary Materials

The following are available online at https://www.mdpi.com/2072-6643/12/12/3835/s1, Table S1: Logistic regression analysis results of model 1, Table S2: Logistic regression analysis results of model 2, Table S3: Logistic regression analysis results of model 3, Table S4: Outcome of logistic regression analysis, Table S5: OR of additive interaction.
