# Supplementary material for: The Association between Purine-Rich Food Intake and Hyperuricemia: A Cross-Sectional Study in Chinese Adult Residents
Source: Nutrients. 2020 Dec 15;12(12):3835. doi: 10.3390/nu12123835 (PMC7765492; doi:10.3390/nu12123835)
Supplement: Supplementary file 1 [file nutrients-12-03835-s001.pdf]

Supplementary Materials

Table S1: Logistic regression analysis results of model 1

Variable	Intake (g/day)		OR, 95% CI (10^{-1}g/day)	P
	Hyperuricemia	Non-hyperuricemia		
Animal derived food	167.53	135.33	1.025（1.019，1.030）	<0.0001
Legumes	70.79	60.72	1.014（1.007，1.022）	0.0003

OR: Odds ratios; 95%CI: 95% confidence intervals.

Table S2: Logistic regression analysis results of model 2

Variable	Hyperuricemia	Non-hyperuricemia	OR, 95%CI	P
**Adjusted**				
Dark vegetables	122.77	118.13	1.001（0.995，1.007）	0.7301
Refined grains	359.74	371.93	0.993（0.989，0.997）	0.0019
**Animal derived food**	**167.53**	**135.33**	**1.025（1.019，1.031）**	**<0.0001**
**Legumes**	**70.79**	**60.72**	**1.015（1.008，1.023）**	**<0.0001**

OR: Odds ratios; 95%CI: 95% confidence intervals.

Table S3: Logistic regression analysis results of model 3

Variable	Hyperuricemia	Non-hyperuricemia	Multivariable OR, 95%CI	P
Gender (Male: Female)	676:435	2542:3160	1.772（1.465，2.143）	<0.0001
Age				
<45: ≥60	311:397	2059:1567	0.806（0.659，0.985）	0.0352
45-59: ≥60	403:397	2076:1567	0.806（0.677，0.959）	0.0151
Region (Rural: Urban)	669:442	3917:1785	0.799（0.690，0.926）	0.0029
Education level				
None: University or college degree	260:66	1328:254	1.058（0.751，1.491）	0.7459
Grad from primary: University or college degree	203:66	1112:254	0.928（0.661，1.302）	0.6640
Lower middle school: University or college degree	345:66	1933:254	0.862（0.629，1.181）	0.3548
Upper middle school: University or college degree	140:66	678:254	0.905（0.641，1.279）	0.5731
Technical or vocational degree: University or college degree	97:66	397:254	1.009（0.698，1.459）	0.9626
BMI				
<18.5: ≥24	34:630	405:2101	0.317（0.219，0.460）	<0.0001

≥18.5& <24: ≥24	447:630	3196:2101	0.516（0.449，0.594）	<0.0001
Alcohol consumption (Drinker: None)	480:631	1794:3908	1.221（1.035，1.422）	0.0182
Hypertension (None: Patient)	602:509	4116:1586	0.572（0.493，0.664）	<0.0001
Diabetes (None: Patient)	1055:56	5548:154	0.921（0.660，1.286）	0.6294
Smoking status (None: Smoker)	675:436	4010:1692	0.999（0.837，1.193）	0.9921
Dietary factors（averages, g/day）				
Dark vegetables	122.77	118.13	1.003（0.997，1.010）	0.2730
Refined grains	359.74	371.93	0.991（0.986，0.995）	0.0001
**Animal derived food**	**167.53**	**135.33**	**1.024（1.018，1.030）**	**<0.0001**
**Legumes**	**70.79**	**60.72**	**1.011（1.003，1.019）**	**0.0080**

BMI: body mass index; OR: Odds ratios; 95%CI: 95% confidence intervals.

Table S4: Outcome of logistic regression analysis

Project	β	P	OR, 95%CI
**Dark vegetables & Animal derived food**			
Dark vegetables	0.003	0.990	1.003（0.644，1.562）
Animal derived food	0.495	0.017	1.641（1.091，2.466）
Dark vegetables * Animal derived food	0.000	0.999	1.000（0.766，1.305）
Constant	-2.400	0.000	0.091
**Refined grains & Animal derived food**			
Refined grains	-0.192	0.387	0.826（0.535，1.274）
Animal derived food	0.403	0.053	1.497（0.995，2.252）
Refined grains * Animal derived food	0.064	0.630	1.066（0.821，1.386）
Constant	-2.118	0.000	0.120

OR: Odds ratios; 95%CI: 95% confidence intervals.

Table S5: OR of additive interaction

Variable	OR	Lower limit	Upper limit
**Dark vegetables & Animal derived food**			
Dark vegetables	1.000	1.000	1.000
Animal derived food	1.000	1.000	1.000
Dark vegetables and Animal derived food	1.000	0.769	1.301
**Refined grains & Animal derived food**			
Refined grains	1.000	1.000	1.000
Animal derived food	1.000	1.000	1.000
Refined grains and Animal derived food	0.938	0.721	1.220

OR: Odds ratios; 95%CI: 95% confidence intervals.
